# A novel ABA functional analogue B2 enhances drought tolerance in wheat

**DOI:** 10.1038/s41598-019-39013-8

**Published:** 2019-02-27

**Authors:** Yuyi Zhou, Rui He, Yuling Guo, Keke Liu, Guanmin Huang, Chuanxi Peng, Yiguo Liu, Mingcai Zhang, Zhaohu Li, Liusheng Duan

**Affiliations:** 1 State Key Laboratory of Plant Physiology and Biochemistry, Engineering Research Center of Plant Growth Regulator, Ministry of Education & College of Agronomy and Biotechnology, China Agricultural University, No 2 Yuanmingyuan Xi Lu, Haidian District, Beijing, 100193 China; 2 College of Agronomy, Qingdao Agricultural University, Qingdao, 266109 China

## Abstract

Drought stress negatively affects wheat growth and yield. Application of drought agent is an effective way to improve crop drought tolerance, therefore increasing crop yield. Based on the structure of abscisic acid (ABA), Pyrabactin and coronatine (COR), we designed the target compound B2. To investigate the function of B2 in alleviating drought stress on wheat, the drought-resistant variety ND212 and drought-sensitive variety LX99 were used under hydroponic conditions. The results showed that B2 had a similar function with ABA, especially 0.01 μmol·L^−1^ B2. Under drought stress conditions, 0.01 μmol·L^−1^ B2 increased the water content of wheat, enhanced the osmotic adjustment ability of leaves, and reduced the toxicity of reactive oxygen species on cells. What’s more, 0.01 μmol·L^−1^ B2 improved the expression level of ABA-responsive genes *TaSnRK2*.*4* and *TaMYB3R1*. It also improved the expression level of drought-responsive genes *TaSRHP* and *TaERF3*. Taken together, B2 enhanced drought tolerance in wheat by activating ABA signaling pathway.

## Introduction

Wheat provides daily sustenance for a large portion of world’s population. It is produced in a wide range of climates and different regions with frequent and various stresses. Drought is one of the major abiotic stresses and the primary cause of yield reduction in crops. It has been estimated to cause an average yield loss of more than 50% for major crops^1,2^. Therefore, establishing an adaptive crop production system in drought soils has been widely focused on.

Hormone regulators was largely explored to breed adaptive varieties, which could enhance the tolerance of crops faced with the soil drought. Previous studies showed that ABA is a plant stress hormone which accumulated under drought stress^3^ and mediated many responses of other abiotic stress^4^. ABA pre-treatment further increased the endogenous ABA level in maize seedling by gas chromatography^5^. Moreover, after pre-soaking seeds with ABA, the activities of antioxidant enzymes, such as superoxidedismutase (SOD), peroxidase (POD), catalase (CAT), ascorbate peroxidase (APX) and glutathione reductase (GR) were significantly enhanced in maize seedlings^6^. Similarly, it was found that the relative water content (RWC) of plants treated with ABA was higher than that of control plants under drought stress. In addition, application of exogenous ABA under water stress accelerated the accumulation of osmolytes, and improved the water status of grains which resulted in higher grain weight in susceptible wheat cultivars^7^. Current research findings indicate that plant hormones regulate many aspects of plant growth, development and the responses to biotic and abiotic stresses. These hormones do not act alone, but interrelated by synergistic or antagonistic cross-talk, so that they can modulate each other’s biosynthesis or responses^8^.

However, the notable shortcomings of ABA are mainly rapidly metabolism and light-induced isomerization^9^, render ABA inactive both *in vivo* and *in vitro*. Thus, structural modification of natural ABA is an inevitable choice. Pyrabactin specifically binded to *PYR/PYL* family proteins, and then inhibited seed germination by reducing the activity of type 2C phosphatases^10^. This compound shares the same receptor with ABA, indicating that it is an structural and functional analogue of ABA, but it has been reported in the literature that Pyrabactin did not induce plant stress-resistance^10,11^. Therefore, the transformation of the skeletal structure of Pyrabactin and the design and synthesis of plant growth regulators with ABA-like functions are of great significance in solving the problems of expensive and easily deactivated ABA. Coronatine, produced by *Pseudomonas coronafacience var*. *atropurpúrea*, is a toxin which induces chlorosis on the leaves of Italian ryegrass^12^. It can increase defense-related protease inhibitors and secondary metabolites, such as volatiles, nicotine, and alkaloid. Also, it plays an important role in resistance to abiotic stress, such as salinity stress^13,14^. In our study, we found that the amide structure of COR and the sulfonamide-structure of Pyrabactin have some similarities. Therefore, we adopted subactive structure splicing method, integrating ABA, Pyrabactin and coronatine, then synthesized compound B2 (Fig. S1). To determine whether B2 has the similar effects with ABA on drought tolerance, two different winter wheat cultivars were used to explore the potential use of B2 in enhancing drought tolerance based on the physiological changes and different expression level of related genes under drought stress conditions.

## Results

### B2 changed wheat morphology under drought stress

**Figure 1:**
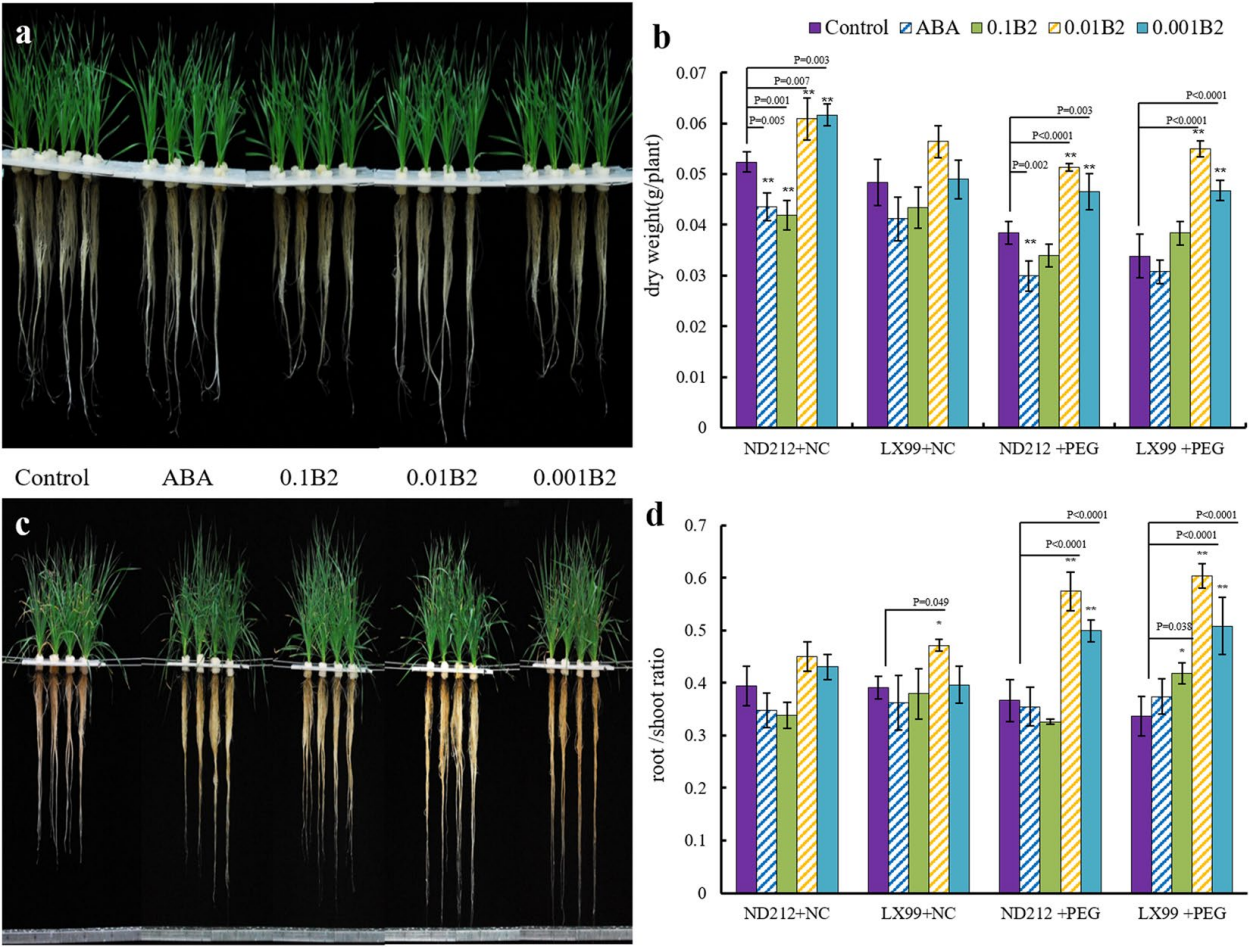
Effect of B2 on winter wheat seedlings. (**a**) Phenotypes of ND212 (right) and LX99 (left) under normal conditions (NC). (**b**) Dry weights of wheat. (**c**) Phenotypes of ND212 and LX99 under drought stress. The wheat in (**a**,**c)** were treated from left to right as controls, ABA, 0.1 μmol·L^−1^ B2, 0.01 μmol·L^−1^ B2 and 0.001 μmol·L^−1^ B2. (**d**) The root/shoot ratio of two cultivars. Values are mean ± SD of three replicates and asterisks denote Student’s test significance compared with the wild type (Tukey, *P < 0.05; **P < 0.01).

The growth of wheat roots with ABA and B2 treatment was significantly affected after five days. The root elongation rate of wheat treated with 0.01 μmol·L^−1^ B2 was much faster than that of other treatments (Fig. 1a). Seedling morphology of ND212 and LX99 with ABA and B2 treatments significantly changed compared with the control after 10 days under drought stress conditions. The leaves of control plants turned yellow, wilted and dehydrated under drought stress, while the plants with ABA and B2 treatment grew much better (Fig. 1c).

At the same time, statistical analysis revealed that there was significant difference among different treatments in dry weight and root/shoot ratio of wheat under drought stress conditions. Moreover, the root dry weight of ND212 and LX99 with B2 treatment increased by 33.7% and 52.3% respectively compared with the control plants under stress conditions (Fig. 1b). What’s more, it was not only observed 45.5% increase of ND212 and 52.8% increase of LX99 in root/shoot ratio pretreated with 0.01 μmol·L^−1^ B2 under normal conditions, but also observed significant increase by 27.6% and 27.7% respectively under drought stress conditions (Fig. 1d).

### B2 increased the RWC of wheat leaf

**Figure 2:**
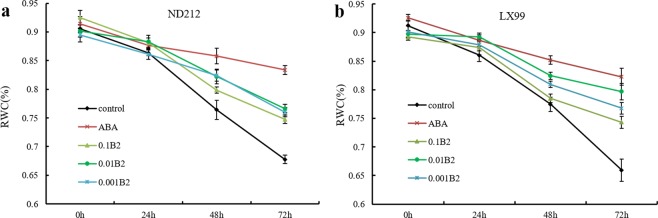
Effect of B2 on relative water content in winter wheat seedlings. (**a**) The relative water content of ND212 changes at 0 h, 24 h, 48 h, and 72 h under drought stress conditions. (**b**) The relative water content of LX99 changes at 0 h, 24 h, 48 h, and 72 h under drought stress conditions. Values are mean ± SD of three replicates.

The relative water content of leaf can reflect plant physiological state after drought stress. As shown in Fig. 2, the leaf RWC of ND212 and LX99 showed a decreasing trend with the extension of drought stress time. But the relative water content of leaves treated with ABA and B2 were always higher than that of control in the range of 0 h to 72 hours (Fig. 2a,b). And the difference was most obviously at 72 hours than any other time. At the same time, the relative leaf water content of ND212 and LX99 treated with ABA and B2 was higher than that of control by 24.7%, 20.8% 13.0% and 12.2%, respectively.

### B2 changed the level of endogenous hormones in wheat leaf

**Figure 3:**
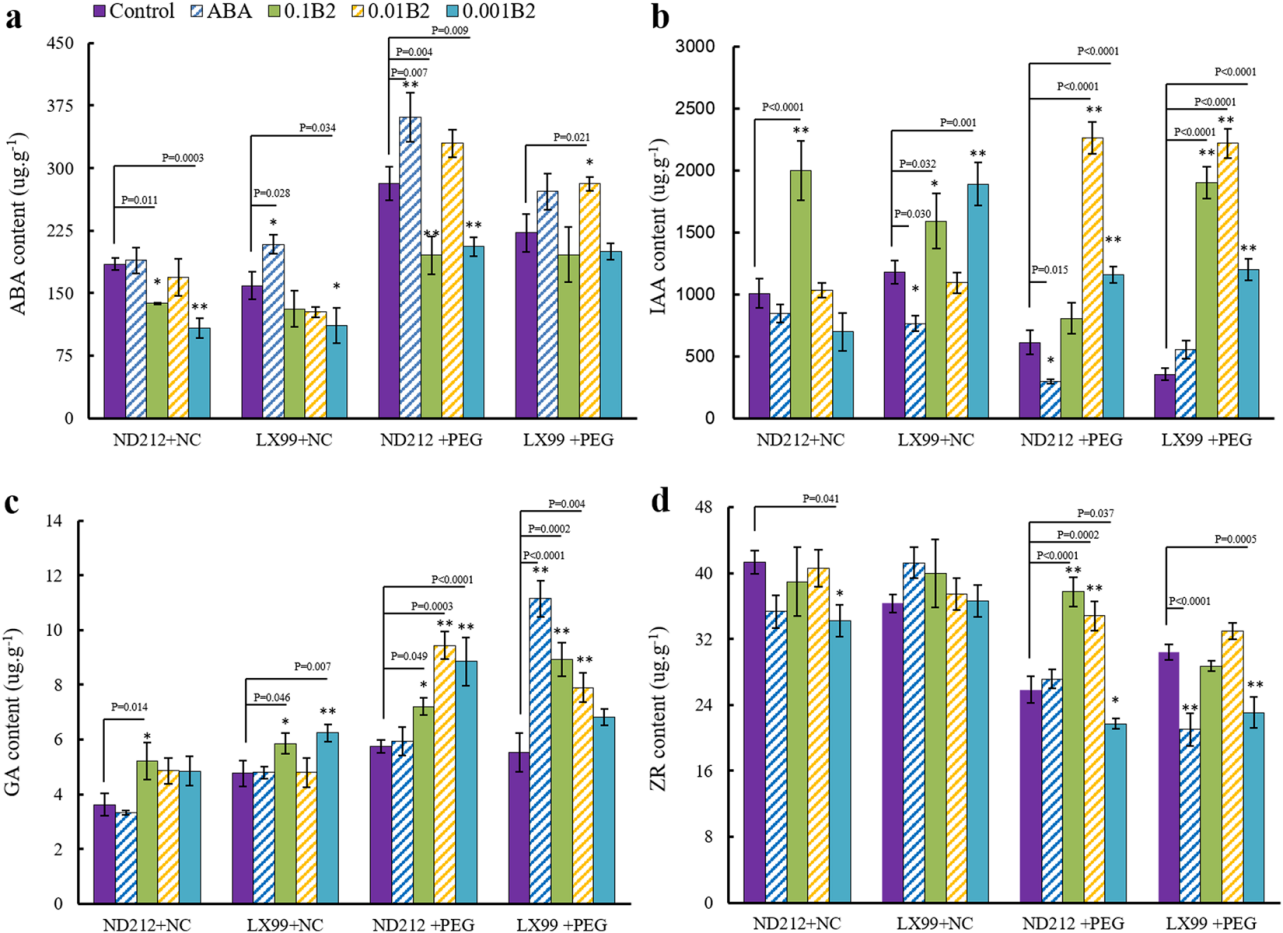
Effect of B2 on content of endogenous hormones in wheat seedlings. (**a**) Evolution of ABA levels. (**b**) Evolution of IAA levels. (**c**) Evolution of GA levels. (**d**) Evolution of ZR levels. Values are mean ± SD of three replicates and asterisks denote Student’s test significance compared with the wild type (Tukey, *P < 0.05; **P < 0.01).

Without drought stress, B2 increased the IAA and GA level, and decreased the ABA level both in ND212 and LX99. While under stress conditions, the level of endogenous hormones has shown a significant interaction between B2 and drought. The content of ABA (Fig. 3a) and GA (Fig. 3c) in leaves with ABA and 0.01 μM B2 pretreated significantly increased compared with that of control. The content of endogenous ABA and GA increased by 78.3% and 64.2% in ND212 respectively, and those in LX99 increased by 76.9% and 42.9% respectively. What’s more, the content of IAA in wheat leaves significantly increased by B2 treatment (Fig. 2b). The IAA content of ND212 and LX99 increased by 124.2% and 87.8% with B2 treatment under drought stress conditions. B2 increased the content of ZR by 34.5% in ND212 (Fig. 3d).

### B2 increased the activity of antioxidant enzymes under drought stress

**Figure 4:**
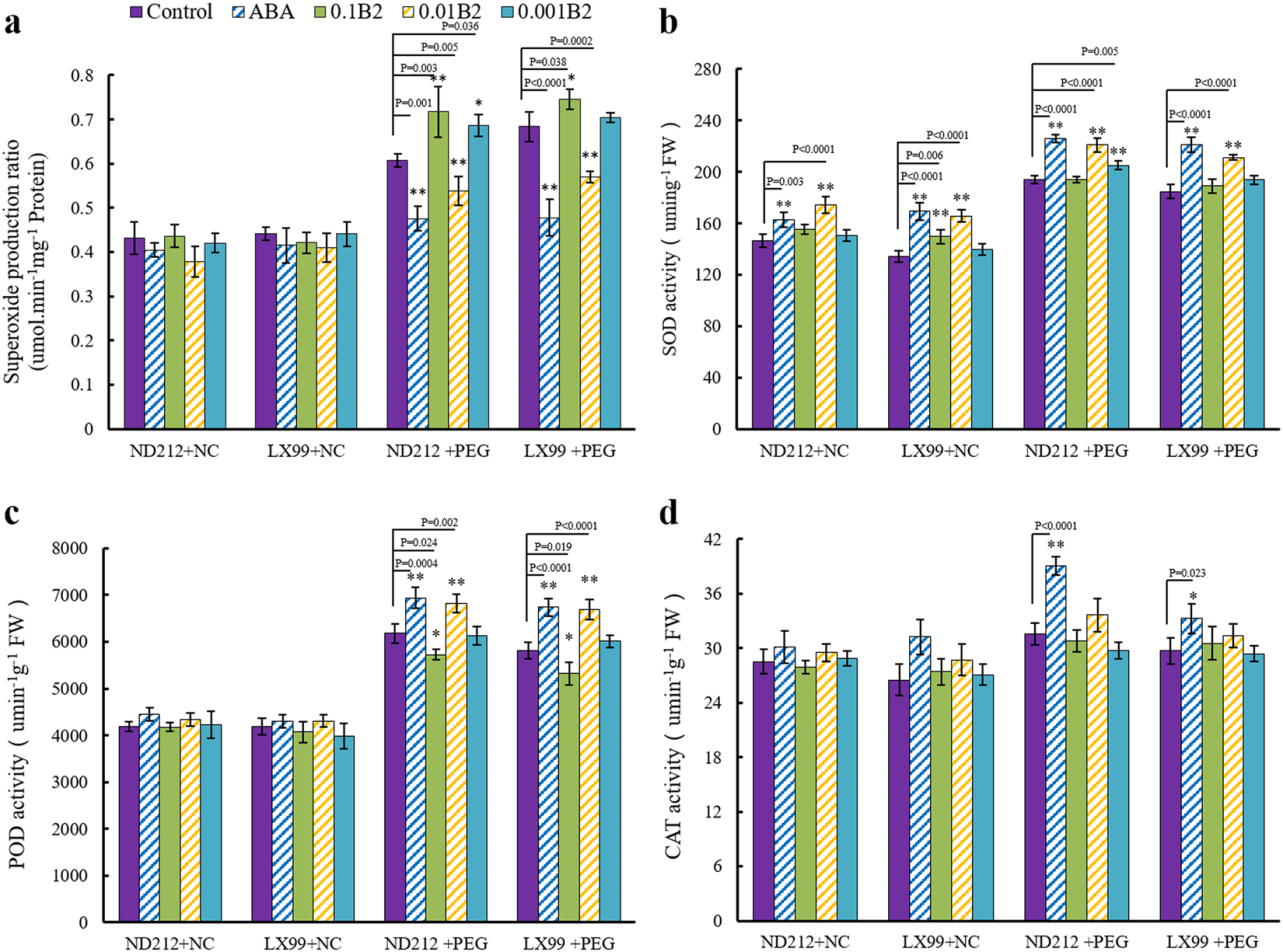
Evolution of superoxide production ratio and enzyme activity levels. (**a**) Superoxide production ratio. (**b**) SOD. (**c**) POD. (**d**) CAT. Values are mean ± SD of three replicates and asterisks denote Student’s test significance compared with wild type (Tukey, *P < 0.05; **P < 0.01).

The production rate of reactive oxygen significantly increased under drought stress (Fig. 4a). The activity of antioxidant enzymes was analyzed after 3 days drought imposition. Activities of antioxidant enzymes SOD were remarkably affected by drought stress. However, B2 increased antioxidant enzymes SOD activity in wheat leaves (Fig. 4b). The level of SOD had shown significant interaction between B2 and drought in both cultivars. The activities of SOD (Fig. 4b), POD (Fig. 4c) and CAT (Fig. 4d) were significantly increased in ND212 by B2. While in LX99, only the SOD and POD activities were significantly enhanced. The activity of CAT was not affected by B2 under drought stress. It can be seen that the decrease of active oxygen may be caused by the increase of SOD, POD and CAT activities.

### B2 improved soluble protein content in wheat leaves under drought stress

**Figure 5:**
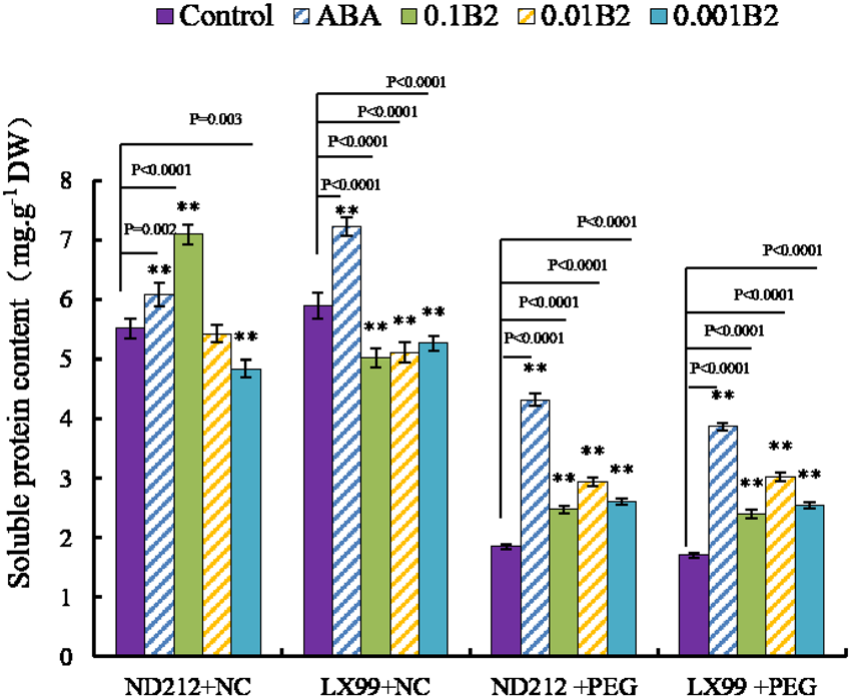
Effect of B2 on soluble protein content in wheat seedlings. Values are mean ± SD of three replicates and asterisks denote Student’s test significance compared with wild type (Tukey, *P < 0.05; **P < 0.01).

After PEG-simulated drought stress, the soluble protein content of wheat leaves was significant higher than that of control, and increased by 1.58 and 1.77-fold in ND212 and LX99 (Fig. 5). However, the soluble protein content decreased after treated with high concentration of B2. Pretreatment with ABA and 0.01 μmol·L^−1^ B2 could effectively increase the soluble protein content of wheat leaf under drought stress conditions.

### B2 enhanced the capacity of photosynthesis under drought stress

**Figure 6:**
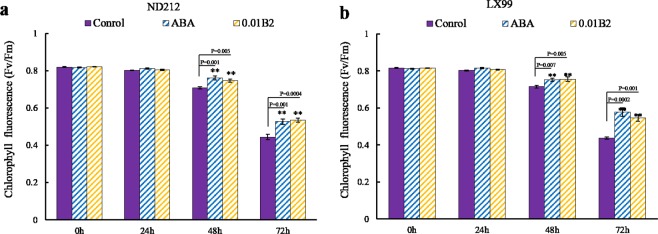
Effect of B2 on the chlorophyll fluorescence in leaves of winter wheat seedlings. (**a**) ND212. (**b**) LX99. Values are mean ± SD of three replicates and asterisks denote Student’s test significance compared with the wild type (Tukey, *P < 0.05; **P < 0.01).

Under adverse conditions chlorophyll fluorescence parameters can effectively monitor the dynamic function of the photosynthetic system of plant leaves^15^. As shown in Fig. 6, ABA and 0.01 μmol·L^−1^ B2 had no significant effect on chlorophyll fluorescence of wheat seedlings under normal conditions, but with the prolongation of drought stress, the chlorophyll fluorescence of wheat gradually decreased. The different treatments reached a significant level after 48 h, and the chlorophyll fluorescence of ND212 (Fig. 6a) and LX99 (Fig. 6b) pretreated with B2 increased by 20.4% and 24.8% respectively. Therefore, ABA and B2 can reduce the rate of decline of chlorophyll fluorescence of wheat under stress conditions, thus keeping it at a higher level.

ABA and B2 pretreatment had no significant effect on photosynthetic rate, stomatal conductance, intercellular CO_2_ concentration, and transpiration rate of wheat seedlings under normal conditions (Table 1). While under drought stress conditions, these indicators decreased significantly. However, contrary to the inhibitory effect of ABA on photosynthetic, the downward trend of photosynthetic indicators could be effectively alleviated by B2. The photosynthetic rate, stomatal conductance, intercellular CO_2_ concentration and transpiration rate of ND212 increased by 13.5%, 15.9%, 23.4%, and 32.6% respectively. Similarly, this indicators of LX99 increased by 15.4%, 23.7%, 21.6%, and 28.4% respectively.

**Table 1:** Effect of B2 on photosynthesis rate (Pn), transpiration rate (Tr), intercellular CO_2_ concentration (Ci) and stomatal conductance (Sc) in winter wheat leaves.

Cultivar	Treatment	Pn (μmol CO_2_/m^2^ per s)	Sc (mol H_2_O/m^2^ per s)	Ci (μmol CO_2_/m^2^ per s)	Tr (mmol H_2_O/m^2^ per s)
ND212 + NC	Control	12.08 ± 0.21	0.084 ± 0.005	185.88 ± 5.58	3.64 ± 0.09
	ABA	12.55 ± 0.44	0.089 ± 0.006	209.15 ± 10.43* P = 0.014	3.52 ± 0.05
	0.01B2	12.40 ± 0.37	0.087 ± 0.003	203.73 ± 10.43	3.58 ± 0.07
ND212 + PEG	Control	7.97 ± 0.28	0.049 ± 0.004	124.68 ± 5.76	1.64 ± 0.04
	ABA	9.19 ± 0.32** P = 0.0004	0.058 ± 0.005* P = 0.049	163.08 ± 5.24** P = 0.0001	1.07 ± 0.09** P < 0.0001
	0.01B2	9.09 ± 0.23** P = 0.001	0.060 ± 0.005* P = 0.019	153.90 ± 6.65** P < 0.0001	1.26 ± 0.05** P < 0.0001
LX99 + NC	Control	11.78 ± 0.40	0.085 ± 0.006	186.65 ± 3.72	3.66 ± 0.05
	ABA	12.39 ± 0.30	0.081 ± 0.004	209.38 ± 1.88* P = 0.023	3.42 ± 0.05** P = 0.0003
	0.01B2	12.26 ± 0.38	0.089 ± 0.006	213.22 ± 9.95* P = 0.011	3.65 ± 0.06
LX99 + PEG	Control	7.80 ± 0.28	0.046 ± 0.004	124.45 ± 7.85	1.69 ± 0.08
	ABA	8.56 ± 0.27* P = 0.01	0.062 ± 0.004** P = 0.001	150.60 ± 4.73** P = 0.001	1.20 ± 0.06** P < 0.0001
	0.01B2	8.99 ± 0.29**P = 0.001	0.059 ± 0.004** P = 0.001	151.28 ± 5.41** P = 0.0004	1.24 ± 0.05** P < 0.0001

Values are mean ± SD of three replicates and asterisks denote Student’s test significance compared with the wild type (Tukey, *P < 0.05; **P < 0.01).

### B2 treatment improved the expression level of *TaSnRK2*.*4*, *TaERF3*, *TaSRHP*, and *TaMYB3R1* which response to drought and ABA

**Figure 7:**
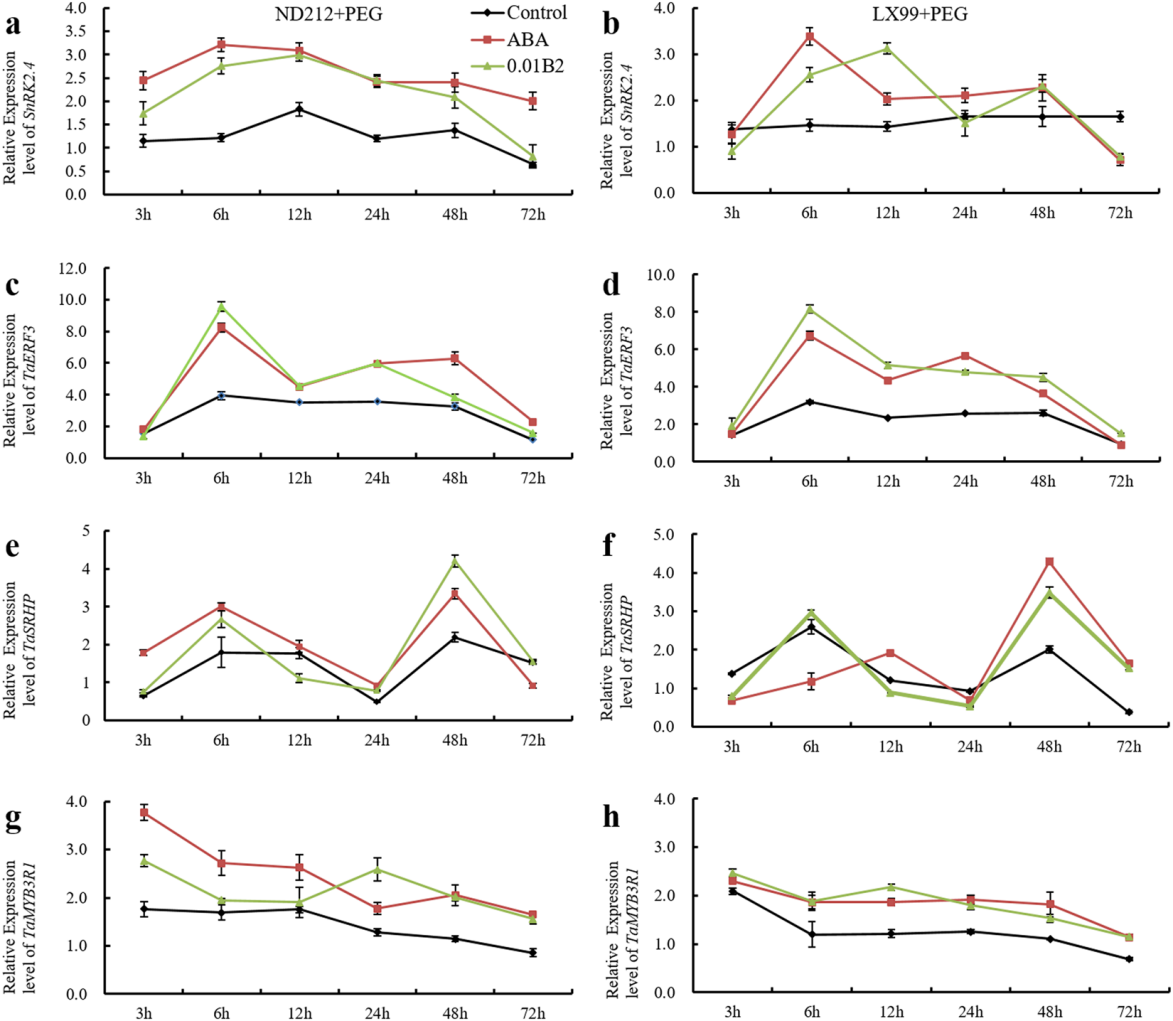
Expression patterns and relative expression level of *TaSnRK2*.*4*, *TaERF3*, *TaSRHP* and *TaMYB3R1* in wheat seedlings at 3 h, 6 h, 12 h, 24 h, 48 h, and 72 h under drought stress conditions.

After ABA or 0.01 μmol·L^−1^ B2 pretreatment, the expression level of these four genes was significantly increased under drought stress. However, the time for each gene up to the highest expression level is different. The expression level of *TaSnRK2*.*4* (Fig. 7a,b) and *TaERF3* (Fig. 7c,d) under drought stress showed an increasing trend first and then decreasing with time. Compared with control, ABA and B2 increased the expression level of *TaSnRK2*.*4* and *TaERF3* in wheat leaves under drought stress. Specifically, B2 increased the expression level of *TaSnRK2*.*4* in ND212 (Fig. 7a) and LX99 (Fig. 7b) by 126% and 73.6% respectively at 6 hours. Similarly, B2 increased the expression level of *TaERF3* in ND212 (Fig. 7c) and LX99 (Fig. 7d) by 143.2% and 155.2% respectively at the same time. In contrast, the expression level of *TaSRHP* showed opposite trend of those genes. B2 increased the expression of *TaSRHP* in ND212 (Fig. 7e) and LX99 (Fig. 7f) by 91.8% and 72.8% at 48 h. B2 improved the expression level of *TaMYB3R1*, and it reached the highest value in ND212 (Fig. 7g) at 24 hours, which was 100.8% higher than that of control. For water-sensitive wheat variety LX99 (Fig. 7h), B2 increased the expression of *TaMYB3R1* by 78.8% at the 12 h.

## Discussion

Drought is one of the major constraints limiting crop growth^16^. Previous studies has demonstrated that ABA plays critical roles in regulating plant growth, development, and response to abiotic stresses such as drought, salt, and cold^17^. In this study, we investigated the effects of the ABA functional analogue B2 on wheat seedling growth, photosynthesis, osmotic adjustment ability, ROS, endogenous hormone, antioxidant enzyme activity, and related genes in wheat under drought stress.

RWC is used extensively to determine the water status of plant relative to their turgid condition. It was proposed that the use of RWC and consumptive use by wheat varieties was as a screening test for drought tolerance under no-irrigated conditions^18^. In this study, we found that ABA and B2 significantly increased the RWC of both wheat cultivars (Fig. 2). Further study showed B2 could promote the roots growth and increased the root/shoot ratio (Fig. 1), thus enhanced the water absorption capacity of wheat seedlings as reported previously^19^.

To reduce the oxidative damage of reactive oxygen species (ROS), plants can adjust the activities of antioxidant system to improve their resistance to drought stress. In this case, the level of antioxidant enzymes, such as SOD, POD, and CAT would increase which was usually used to characterize the antioxidant capacity^20,21^. Among the antioxidant enzymes, SOD can convert •O_2_^−^ to H_2_O_2_ and then H_2_O_2_ was further converted to O_2_ and H_2_O by antioxidant enzymes such as CAT and POD. Previous studies have demonstrated that the POD activity was enhanced by a small molecule ABA mimic (AM1)^11^. It has been reported that exogenous application of ABA significantly increased the activities of SOD, CAT, POD and glutathione reductase (GR) and increased the content of ascorbate, but reduced glutathione, a-tocopherol and carotenoid^22^. In this study, B2 increased the SOD, POD, and CAT activities in the wheat seedlings remarkably under drought stress (Fig. 4). These results implicated that B2, just as ABA, could improve the ability of scavenging ROS via raising the activities of antioxidant enzymes in wheat seedlings under drought stress, thus B2 could reduce oxidative damages. These maintenance phenomena could also be confirmed via the increase in soluble protein level due to the positive correlations among SOD, CAT and soluble protein (Fig. 5), which increased the osmotic adjustment abilities of wheat seedlings.

ABA is an important signal factor in response to dehydration and it can regulate the water status of plant via stomatal conductance and induce genes involved in dehydration resistance^23–26^. It was reported that the synthesis of ABA was enhanced under drought stress. ABA could induce plant antioxidant defense system and suppress ROS damages under drought stress. Previous studies have also demonstrated that avoidance mechanism induced by water-stress was dependent on ABA synthesis. ABA could activate some enzymes, regulate the osmotic adjustment^24^, and improve the hydraulic conductivity of roots via inducing gene expression of aquaporin family^25^. In our study, the endogenous ABA level was improved under drought stress, and it was also further improved after treated with B2 (Fig. 3a). These results suggested that B2 promoted ABA accumulation in plants. It helped to regulate the antioxidant enzyme defense system and enhanced its osmotic adjustment abilities, therefore reducing oxidative damages of ROS.

It was found that many genes in wheat, such as *TaSnRK2*.*4*, *TaMYB3R1*, *TaSRHP* and *TaERF3*, their expression level has changed obviously under abiotic stress conditions. *TaSnRK2*.*4*, was identified in common wheat, activated by osmotic stress. It was indicated that *TaSnRK2*.*4* was much sensitive to NaCl and drought stresses, and less sensitive to ABA^27^. In this study, the expression level of *TaSnRK2*.*4* rapidly reached a high level at 3 h and continued to accumulate up to 6 h by B2. *TaMYB3R1*, a new member of wheat *MYB* gene family, played a vital role in regulating the response to cold, salt and drought stress^28^, as reported previously in rice^29^. In addition, it was also found that *TaMYB3R1* transcript expression was induced following treatment with ABA^28^. Over-expression of ABA-responsive gene *TaSRHP* in *A*. *thaliana* resulted in enhancing resistance to salt and drought stresses. And the sensitivity of the transgenic *A*. *thaliana* to ABA was also increased compared with that of wild-type^30^. Our study showed that the expression of *TaSRHP* (Fig. 7e,f) was notably increased at 48 h after drought stress. We could infer that *TaSRHP* might play a regulatory role after *TaSnRK2*.*4* (Fig. 7a,b). *TaERF3* (Fig. 7c,d) positively regulated wheat adaptation response to salt and drought stresses through the activation of stress-related genes. The accumulation level of both proline and chlorophyll was significantly increased in *TaERF3* over-expressing plants under salt and drought stress. Further study found that *TaERF3*-silencing of wheat generated through virus-induced gene silencing method displayed more sensitive to salt and drought stresses compared with the control plants^31^. In this study, we found that the expression level of *TaERF3* pre-treated with ABA and B2 was much higher than control plants at 6 h after drought stress. It can be seen that *TaERF3* and *TaSnRK2*.*4* played a regulatory role in the early stages of drought stress.

Drought stress severely inhibited the photosynthesis of plant leaves through causing stomatal closed, water imbalanced, and enzymatic activity decreased in the Calvin cycle^32^, therefore inhibiting plant growth or even killing them ultimately. Photosynthesis is an important indicator of plant growth. Drought significantly reduces plant photosynthetic capacity (Table 1). In this study, B2 significantly enhanced the photosynthesis of wheat leaves, while ABA had no obvious effect on that (Table 1). It suggested that B2 might play a special regulatory mechanism.

The drought response of plant is regulated in a complex way through the interaction of various endogenous hormones^33^. Therefore, endogenous hormones in plants play an important role in resisting drought stress. IAA, ZR and GA content in leaves were decreased under water stress (Fig. 3b–d). Pre-sowing seed treated with B2 partially overcame this trend. However, it is interesting that, under drought conditions, there was no consistent effect between B2 and ABA on endogenous hormones IAA, GR and ZA. It indicated that B2 has an unknown potential to regulate endogenous hormone levels in wheat.

In summary, B2, an ABA functional analogue, enhanced the drought resistance in wheat by improving the osmotic adjustment ability and enhancing the active oxygen scavenging capacity through increasing antioxidant enzyme activity. It also significantly enhanced the photosynthesis of wheat leaves. In addition, B2 owns a unique regulatory mechanism, just as significantly increasing the endogenous hormone content of wheat under drought stress conditions. Our results may provide an economic method to solve drought stress on field crops.

## Methods

### Plant material and stress treatments

The full wheat seeds were selected, and soaked in the distilled water at 25 °C for 24 hours, then spread in the plastic pots with clean quartz sand. Seedling sat one-leaf stage were transplanted into the plastic containers containing Hoagland’s solution for further culture in greenhouse (light: dark 14:10) with 50% humidity at 22/25 °C (light/dark) under light supply of 400 mol· (m^2^∙s)^−1^. Two wheat cultivars were planted at the same hydroponic box, in which the left side was Liangxing99 (LX99) and the right side was Nongda212 (ND212). Each kind wheat planted 16, and each treatment was set with 4 replicates.

After 48 hours, wheat seedlings were treated with ABA and B2 by adding to the nutrient solution respectively. The ABA concentration was set to 0.01 μmol·L^−1^ (ABA) and B2 was 0.1 μmol·L^−1^ (0.1B2), 0.01 μmol·L^−1^ (0.01B2) and 0.001 μmol·L^−1^ (0.001B2) by preliminary experiment. The seedlings at two-leaf stage were transferred to Hoagland’s total nutrient solution containing 20% PEG 6000 for simulated drought situation. All plant samples, including normal conditions (NC) and PEG treatment, were collected three biological replicates at required time points and used for physiological measurements or molecular detections.

### Physiological measurements

After ABA and B2 treatment, the leaf RWC was measured on the youngest fully expanded leaves following the method of Turner^34^ at 0, 24, 48 and 72 hour. And gas exchanges were measured at 72 h after treated with ABA and B2 using a LI-6400 infrared gas analyzer (LICOR, Lincoln, NE, USA), operated with a 6400-02 LED light source (LICOR) providing 400 μmol/m^2^ per sPPFD. The primary CO_2_ concentration in the chamber was 390 μmol/mol. Eight plants were examined per replication.

After treated with ABA and B2 for 72 hours, ·O_2_^−^ was measured as described in Wang *et al*.^35^. 0.5 g Leaf tissues was homogenized in an ice bath in 4.0 mL of 50 mM sodium phosphate buffer (pH = 7.8) and centrifuged at 12,000 g for 15 min at 4 °C. The supernatant was immediately assayed for ·O_2_^−^. A standard curve with NO_2_^−^ was used to estimate the production rate of ·O_2_^−^. The content of soluble proteins was determined using the method of Bradford^36^, based on a standard curve pre-established with bovine serumalbumin. Leaf tissue (0.5 g) was homogenized in 4 mL of 50 mM sodium phosphate buffer (pH = 7.0), 0.1 mM EDTA-Na_2_, 1 mM L-isoascorbic acid, 1% (w/v) PVP, and 0.05% (w/v) Triton X-100 in an ice bath. The homogenate was centrifuged at 12,000 g for 15 min at 4 °C. The resulting supernatant was used for assays of the activities and staining of SOD, CAT, and POD, according to the procedure described in Parida *et al*.^37^.

### Quantification of Hormones by ELISA

The methods for IAA, ABA, GA and ZR extraction and purification, were modified from those described by Bollmark *et al*.^38^ and Yang *et al*.^33^.The mouse monoclonal antigens and antibodies against IAA, ABA, GA and ZR, and Ig-Ghorseradish peroxidase used in ELISA were produced at the Phytohormones Research Institute China Agricultural University. The specific monoclonal antibody and other possible nonspecific immuno reactive interference were checked previously and proved reliable^33^. The results are the means ± SE of at least four replicates.

### RNA extraction and quantitative RT-PCR

Total RNAs of leaves were extracted by Easy Pure Plant RNA Kit (TransGene Biotech, Beijing) and then determined using Nano-Drop2000 spectrophotometer. First-strand cDNA was prepared using SuperScript III RNase H–Reverse Trancrip-tase (Invitrogen, USA) using 2 μg of total RNA. Quantitative RT-PCR procedures were carried out by the Light Cycler System (Bio-Rad, Richmond, CA) with the SYBR^®^ Premix Ex TaqTM Kit (TaKaRa, Dalian, China), in a 15 μL reaction solution containing 7.5 μL of SYBR^®^ Premix Ex TaqTM 2×, 0.3 μmol·L^–1^ of each forward and reverse primers (Table S1), and 1.5 μL of cDNA template, and appropriate amounts of sterile ddH_2_O. All qRT-PCR amplification conditions were standardized according to the manufacturer’s instructions. Three independent replicates were performed on each cDNA sample. The relative expression level was calculated using the 2^−△△Ct^ formula^39^ with the reference genes *β-actin* gene (GenBank ID: AB181991.1) as internal standard.

## Supplementary information

Figure S1 and Table S1

## Data Availability

The datasets generated and analyzed during the current study are available from the corresponding author on reasonable request.
