# Supplementary material for: A novel ABA functional analogue B2 enhances drought tolerance in wheat
Source: Sci Rep. 2019 Feb 27;9:2887. doi: 10.1038/s41598-019-39013-8 (PMC6393525; doi:10.1038/s41598-019-39013-8)
Supplement: Supplementary file 1 — Figure S1 and Table S1 [file 41598_2019_39013_MOESM1_ESM.docx]

**Research Article**

**A novel ABA functional analogue B2 enhances drought tolerance in wheat**

Yuyi Zhou^1+^, Rui He^1+^, Yuling Guo^1^, Keke Liu^1^, Guanmin Huang^1^, Chuanxi Peng^1^, Yiguo Liu^2^, Mingcai Zhang^1^, Zhaohu Li^1^, Liusheng Duan^1^*

^1^State Key Laboratory of Plant Physiology and Biochemistry, Engineering Research Center of Plant Growth Regulator, Ministry of Education &College of Agronomy and Biotechnology, China Agricultural University, No 2 Yuanmingyuan Xi Lu, Haidian District, Beijing 100193, China

^2^College of Agronomy, Qingdao Agricultural University, Qingdao 266109, China

+ These two authors have equal contributions

**Corresponding author:** Liusheng Duan, State Key Laboratory of Plant Physiology and Biochemistry, Engineering Research Center of Plant Growth Regulator, Ministry of Education & College of Agronomy and Biotechnology, China Agricultural University, No 2 Yuanmingyuan Xi Lu, Haidian District, Beijing 100193, China; Phone: +86 18601272095; E-mail:[18601272095@163.com](mailto:18601272095@163.com)

**Figure S1**


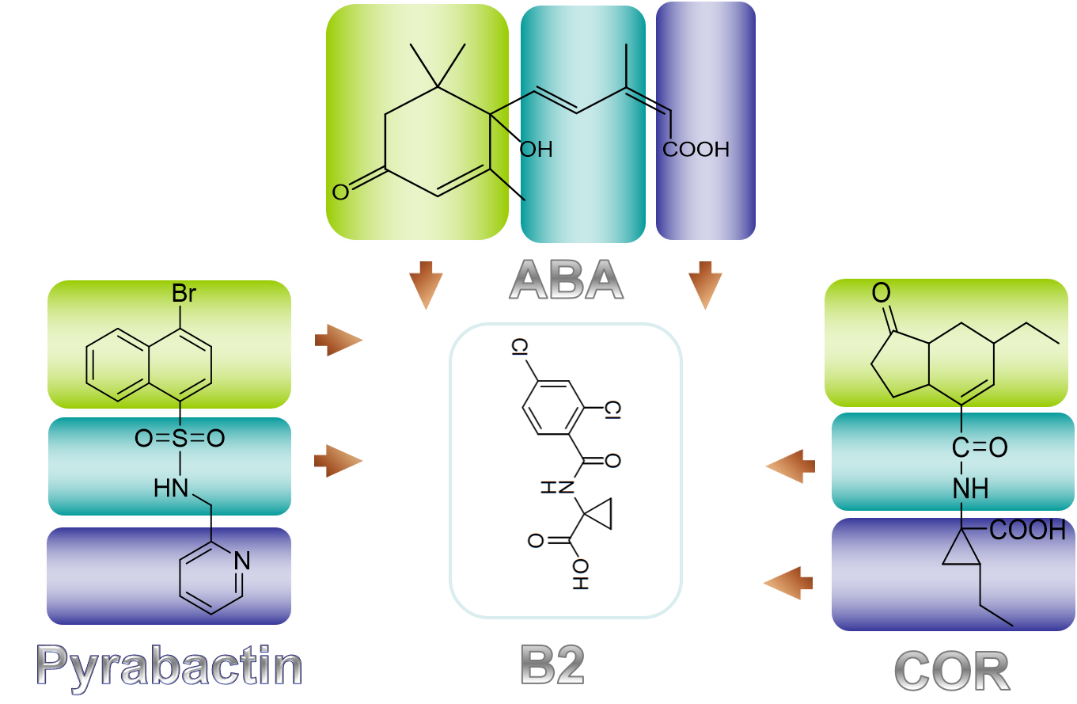


**Figure S1. The synthesis process of B2.** We adopted subactive structure splicing method, integrating ABA, Pyrabactin and Coronatine, and then synthesized compound B2

**Table S1. Gene-specific primers used in this research**

| Gene | primer |
| --- | --- |
| *β-actin* | Forward: 5’-TGCTATCCTTCGTTTGGACCTT-3’  Reverse: 5’-AGCGGTTGTTGTGAGGGAGT-3’ |
| *TaSnRK2.4*  *TaMYB3R1* | Forward: 5’-GGTTCATGCAAGCGGAGAGC-3’  Reverse: 5’-AACCAAAACCAAACAGAAGCAAAC-3’  Forward: 5’-CAGGTGTCGGTCTTTCTCGTTT-3’  Reverse: 5’- GTGGTCAATGGCTCTGCTGTTA-3’ |
| *TaSRHP*  *TaERF3* | Forward: 5’- CGGCCGCGACACCTTCATA-3’  Reverse: 5’- GGTCGCCGGGAAGCACTT-3’  Forward: 5’- AGCAATCAGGCAAAGCAACC-3’  Reverse: 5’- ACGACTCAGAAGGAACCACGAC-3’ |
